# Enantioselective Crystal Growth Induced by Mesoscopic Helical Platforms

**DOI:** 10.1002/chir.70120

**Published:** 2026-07-03

**Authors:** Anthony Boudier, Guillaume Raffy, Emilie Pouget, Sylvain Nlate, Dario M. Bassani, Reiko Oda

**Affiliations:** ^1^ Univ. Bordeaux, CNRS, Bordeaux INP, CBMN, UMR 5248 Pessac France; ^2^ Univ. Bordeaux, CNRS, Bordeaux INP, ISM UMR 5255 Talence France; ^3^ WPI‐Advanced Institute for Materials Research Tohoku University Sendai Japan

**Keywords:** chirality induction, enantioselective crystal growth, mesoscopic helical structure

## Abstract

We report enantioselective crystallization of tetraphenylethylene (TPE) directed by chiral silica nanohelices (SNHs), yielding crystalline assemblies with distinct morphologies and chiroptical signatures that are modulated by both template handedness and evaporation kinetics. When TPE solutions are deposited onto SNH‐functionalized substrates and allowed to crystallize through slow solvent evaporation at 4°C, small orthorhombic crystals (2–5 μm) form with strong induced electronic circular dichroism (ECD) and circularly polarized luminescence (CPL) whose sign is correlated to the underlying SNH handedness. In contrast, rapid evaporation at room temperature leads to dendritic crystalline assemblies with strong ECD but weaker CPL with reduced correlation to the template handedness. Fluorescence microscopy confirms that emission originates exclusively from crystalline domains, consistent with restriction of intramolecular rotation upon crystal packing. The results show that right‐handed (*P*‐) and left‐handed (*M*‐)SNH preferentially promote the formation of distinct TPE enantiomeric crystals, providing a robust platform for template‐directed chirality‐controlled crystallization.

## Graphical abstract

Chiral silica nanohelices with controlled handedness (*P* or *M*) act as mesoscopic templates that bias the enantioselective outcome of tetraphenylethylene conglomerate crystallization, preferentially nucleating *M*‐ or *P*‐enantiomorphic crystals through interfacial chirality transfer.

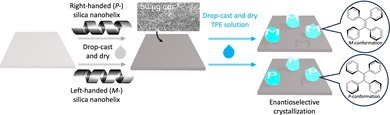

## 1 Introduction

Chirality, the non‐superimposability of an object on its mirror image, is a fundamental organizing principle of matter with consequences spanning weak‐force physics, molecular biology, and materials science. In the pharmaceutical industry, its practical stakes are particularly high: The majority of newly approved drugs are single enantiomers, making enantiopure synthesis and separation a central challenge [1]. Among the various resolution strategies, such as extraction from natural chiral sources or asymmetric synthesis, the separation of racemic mixtures through crystallization‐based resolution remains the most economical and scalable route to enantiopurity [2, 3, 4]. Its principal limitation, however, is thermodynamic: Classical resolution yields at most 50% of the desired enantiomer. Solid‐state deracemization has emerged as a compelling route for complete conversion to a single enantiomer under thermodynamically controlled conditions. Notable examples include Viedma ripening, which shows total symmetry breaking through a nonlinear autocatalytic‐recycling mechanism operating during crystal growth, driving a system containing both enantiomers to homochirality without any chiral reagent [5]. Noorduin et al. demonstrated that circularly polarized (CP) light can act as the initial symmetry breaking input in an amino acid derivative system, resulting in enantiomeric excess, which then is amplified by a grinding process, elegantly coupling a photochemical bias with a mechanical amplification mechanism [6]. More recently, strategies exploiting dynamic conformational isomerism have demonstrated that chiral environments can bias equilibria toward a single enantiomeric conformer, enabling atom‐economical conversions approaching full enantiopurity without stoichiometric chiral auxiliaries [7].

Using tetraphenylethylene (TPE) as a model system, we demonstrate that morphologically chiral silica nanohelices (SNHs) can bias the enantioselective outcome of crystallization of aggregation‐induced emission (AIE) chromophores through interfacial contact alone. Among organic chromophores, TPE is particularly well suited for probing chiral induction during crystallization. In solution, Rouillon and colleagues showed that rapid intramolecular rotation of the C=C bond renders TPE non‐emissive [8]. Emission is activated upon restriction of the rotation in the solid state, effectively suppressing background signal from solution. Similarly, weaker emission from less compact amorphous areas with respect to crystalline domains renders the latter clearly visible by fluorescence [9, 10].

TPE is known to adopt a propeller‐like geometry that interconverts rapidly between two enantiomeric conformers in solution. This interconversion is however hindered upon crystallization, resulting in a rare class of achiral compounds capable of conglomerate crystallization into helical (*P* or *M*) enantiomorphs [11]. The outcome, conglomerate versus racemic packing, predominant handedness, and enantiomeric excess, is highly sensitive to crystallization conditions [12]. Enantiopure TPE crystals with mirror‐image CD signals have been exploited in asymmetric autocatalysis with chirality amplification [13]. Chiral induction in TPE crystallization has been achieved through molecular chiral auxiliaries [14, 15, 16, 17, 18, 19], liquid crystals [20, 21, 22, 23], polymers [24, 25], or gels [26, 27]. At the nanoscale, Tsunega et al. demonstrated that evaporating TPE solutions onto chiral silica nanofibers induces chiral selectivity as revealed by CP luminescence (CPL) [28]. Recently, extended TPE derivatives embedded in crystalline cellulose have been reported to exhibit strong CPL activity [29].

We have previously reported that mesoscopic helical silica structures with controlled handedness (right‐handed [*P*‐] or left‐handed [*M*‐]SNHs) can induce chiroptical signals—induced circular dichroism (ICD) and induced CPL (ICPL)—from various achiral molecules and nanoparticles assembled on their surfaces, including polycyclic aromatics (e.g., pyrene and perylene), porphyrins, gold nanoparticles, and perovskite nanocrystals [30, 31, 32, 33, 34, 35, 36]. More recently, dynamic and racemic mixtures of *M* and *P* foldamers assembled on SNHs exhibited CD and CPL signals, with the transferred chiral memory persisting for several minutes before complete racemization after separation [37]. Analysis of the relaxation profile indicated a weak (~4%) enantiomeric excess of the foldamers, attributed to enantioselective interactions with the SNHs.

**FIGURE 1:**
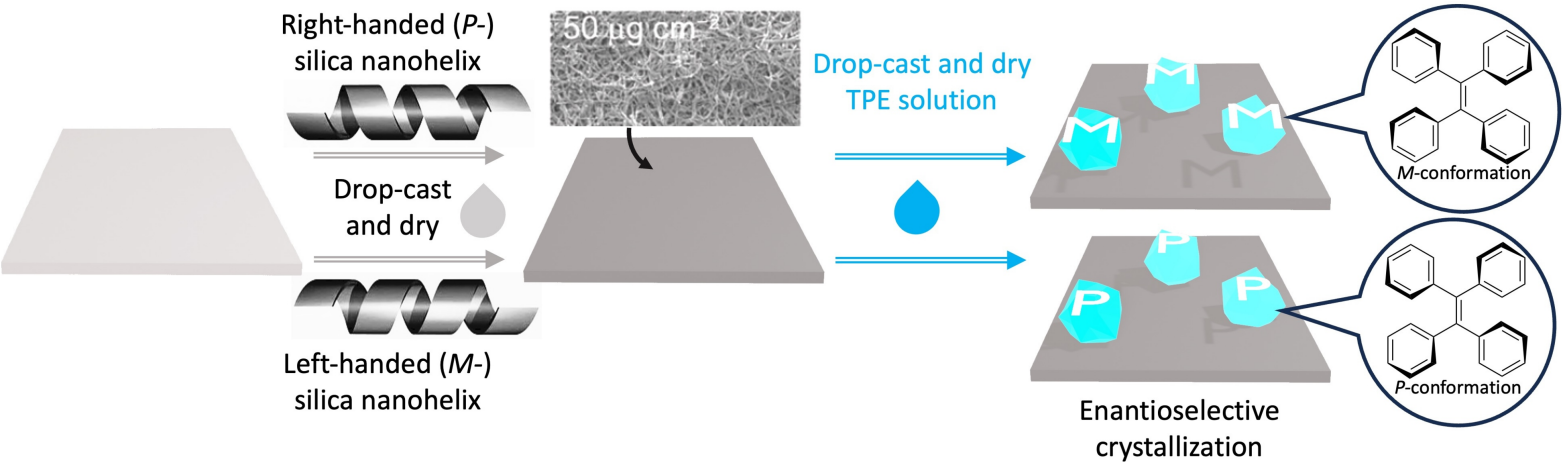
Schematic representation of the preparation of the samples from solutions of TPE drop‐cast onto glass or quartz substrates functionalized with an amorphous film of *M*‐ or *P*‐silica nanohelix.

Here, we show that SNHs can also induce chiroptical signals from TPE upon co‐deposition as thin films. In solution, TPE exists as a very weakly emissive racemic mixture, but crystallization produces strong emission through hindered *E*, *Z* isomerization in the solid. When drop‐cast onto SNH films, TPE exhibits intense CD and CPL signals. Optical microscopy revealed that CPL arises from enantiomeric crystals, whereas CD originates from both supramolecular TPE arrangements at the SNH interface and crystalline domains (schematically shown in Figure 1).

## 2 Materials and Methods

### 2.1 Chemicals

TPE (CAS: 632‐51‐9) was purchased from TCI Europe. Toluene (108‐88‐3), acetonitrile (75‐05‐8), methanol, isopropanol, absolute ethanol, tetraethyl orthosilicate (TEOS), 1‐bromohexadecane (112‐82‐3), *N*,*N*,*N*′,*N*′‐tetra‐methylethylenediamine (TMEDA, 110‐18‐9), silver acetate (563‐63‐3), L‐(+)‐tartaric acid (87‐69‐4), and D‐(−)‐tartaric acid (147‐71‐7) were obtained from Sigma‐Aldrich. All chemicals were used without further purification, except TMEDA, which was centrifuged to remove a white precipitate before use.

### 2.2 Preparation of SNHs

SNHs were synthesized in three steps:

1. **Organic nanohelices**: The synthesis of gemini tartrate surfactant, *N*,*N*′‐dihexadecyl‐*N*,*N*,*N*′,*N*′‐tetramethylethylene diammonium (abbreviated as 16‐2‐16) complexed with tartrate counterions was previously reported [38]. A solution of 16‐2‐16 (3.57 mg in 5 mL of water) was prepared by ultrasonication and dissolution at 60°C for 10 min, cooled, and incubated at 20°C for 4 days with either L‐ or D‐tartrate (yielding right‐handed *P*‐type or left‐handed *M*‐type helices, respectively).
2. **Silica coating:** TEOS (0.5 mL) was mixed with 10 mL of aqueous L‐ or D‐tartaric acid (0.1 mM) at 20°C for 7 h. Then, 5 mL of this solution was added to the organic helices and reacted at 20°C for 20 h to form silica‐coated hybrid nanohelices, which were collected by centrifugation and washed with cold water.
3. **Organic template removal:** The hybrid helices were washed with methanol (3×, 60°C, 10 min) and an isopropanol:ethanol mixture (50:50, 3×, 60°C, 10 min), leading to the formation of *P*‐ or *M*‐SNH, which was then redispersed in the same solvent mixture [39].

### 2.3 Film Preparation

SNH suspensions (200 μg in EtOH:isopropanol 1:1) were drop‐cast on quartz substrates (2 × 2 cm^2^) and dried at room temperature. TPE (1 mM in toluene, 20 μL) was drop‐cast on the SNH film and dried either at 20°C (fast) or 4°C (slow, 24 h).

#### 2.3.1 Electron Microscopy

Sample morphology was characterized by TEM (CM120, Philips) at 120 kV. Samples were prepared by drop‐casting 5 μL of SNH dispersion (0.05 μg/μL in EtOH:isopropanol) onto carbon‐coated copper grids and air‐drying.

#### 2.3.2 Electronic Circular Dichroism (ECD)

ECD and UV–vis spectra were recorded using a CD spectropolarimeter (J‐1500, JASCO) with a solid‐state holder. Diffuse reflectance CD (DRCD) was measured using an integrating sphere (DRCD‐575) attached to the spectropolarimeter. Linear dichroism artifacts were minimized by averaging spectra at 0° and 90° sample orientation.

#### 2.3.3 CPL

CPL and fluorescence spectra were acquired using a CPL spectrophotometer (CPL‐300, JASCO) at 750‐V HT voltage. Samples were excited at 333 nm (8‐nm excitation and emission bandwidths), with emission recorded from 375 to 575 nm.

CD and CPL were measured at two azimuth angles, 0° and 90°, and summed to minimize the LD effect.

#### 2.3.4 Optical Microscopy

Optical and fluorescence images were obtained using a Nikon Eclipse E600FN microscope with a Nikon DXM1200 camera and 10× Plan Fluor objective. Fluorescence images were recorded under 330–380‐nm excitation.

#### 2.3.5 Polarization‐Resolved Fluorescence Microscopy

A homebuilt polarimetric setup [40] was integrated into an Olympus IX71 epifluorescence microscope. An air objective (60×, NA = 0.90) or an oil objective (100×, NA = 1.40) was used to achieve ~300‐ or ~200‐nm resolution, respectively. A mask was placed at the primary image plane of the microscope to reduce the field of view (FOV) by one‐half. A motorized rotating retarder that could be swapped between a half waveplate (superachromatic, Thorlabs) and a quarter waveplate (superachromatic, Bernhard‐Halle), followed by a fixed polarizing beamsplitter, was used to image two copies of the masked FOV side by side on the same camera. Upon rotation of the waveplate, this enabled sequential imaging to produce a polarization‐encoded 3D data cube. Color‐coded images were computed to represent key polarization parameters. Only the azimuthal orientation of the linearly polarized (LPL) component is represented in Figure 5.

## 3 Results and Discussion

**FIGURE 2:**
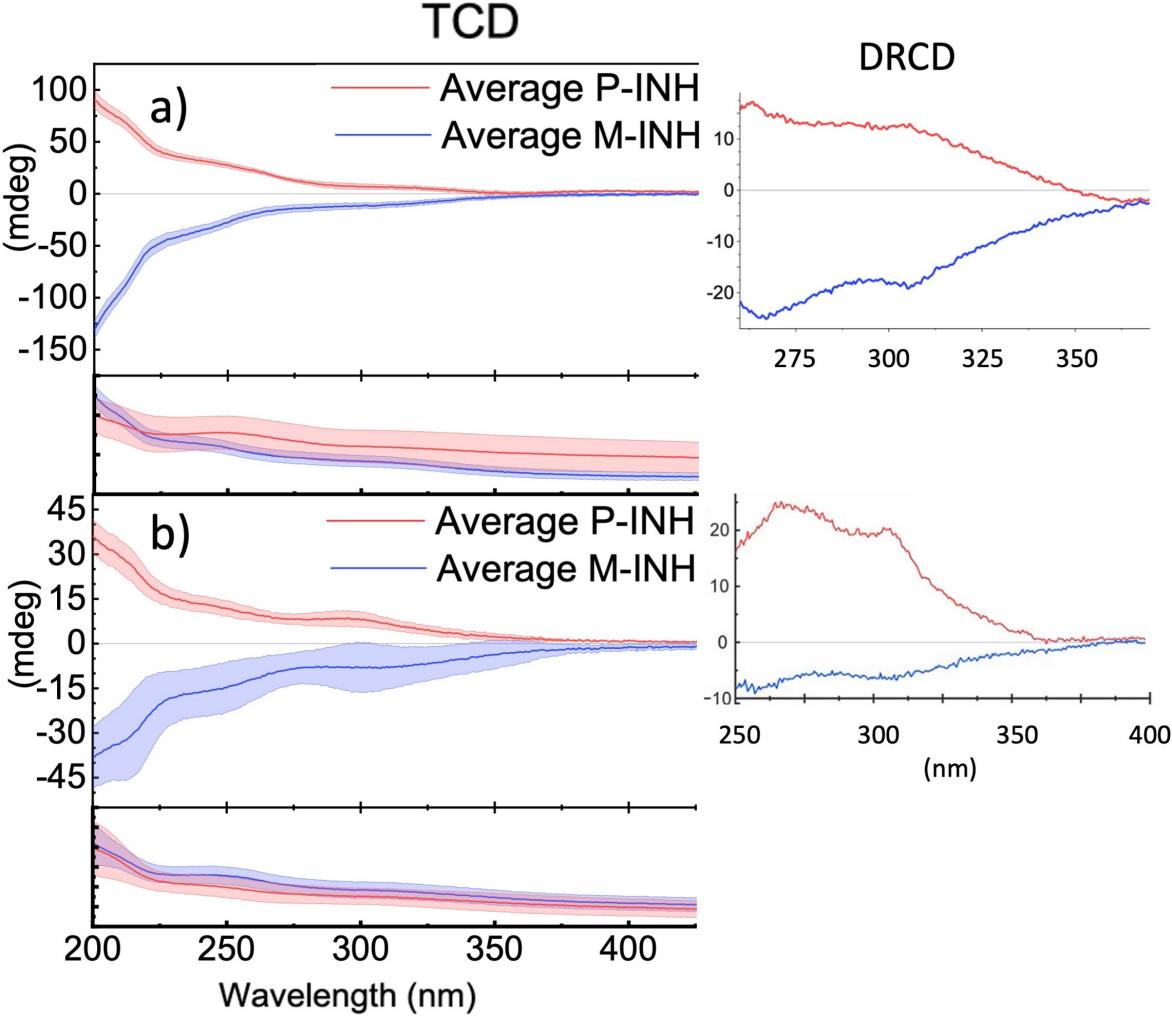
Transmission ECD and DRCD (inset) and absorbance spectra of (a) slow (4°C) and (b) fast (20°C) dried TPE toluene solution on *M*‐ and *P*‐SNH films. Shaded areas indicate spread of signals used in determining the average signal (colored solid line).

In solution, TPE displays a strong phenyl‐centered π–π* absorption band at 250–280 nm and a weaker band near 300–320 nm. DRCD measurements (to remove scattering contributions from SNHs) were performed on the co‐assembled TPE/*P*‐ or *M*‐SNH thin films, which exhibited mirror‐image signatures in the 270–350‐nm region, with shoulders at ~245 and 305 nm (Figure 2). Dissymmetry factors (*g*_abs_) at 305 nm of ~1.5 × 10^−3^ for *P*‐SNH and 3.0 × 10^−3^ for *M*‐SNH were reproducible regardless of the evaporation speed.

Due to fast *E*, *Z* isomerization, TPE is only very weakly emissive in solution but becomes strongly luminescent when dried on SNH‐coated substrates, with an emission maximum at 435 nm.

**FIGURE 3:**
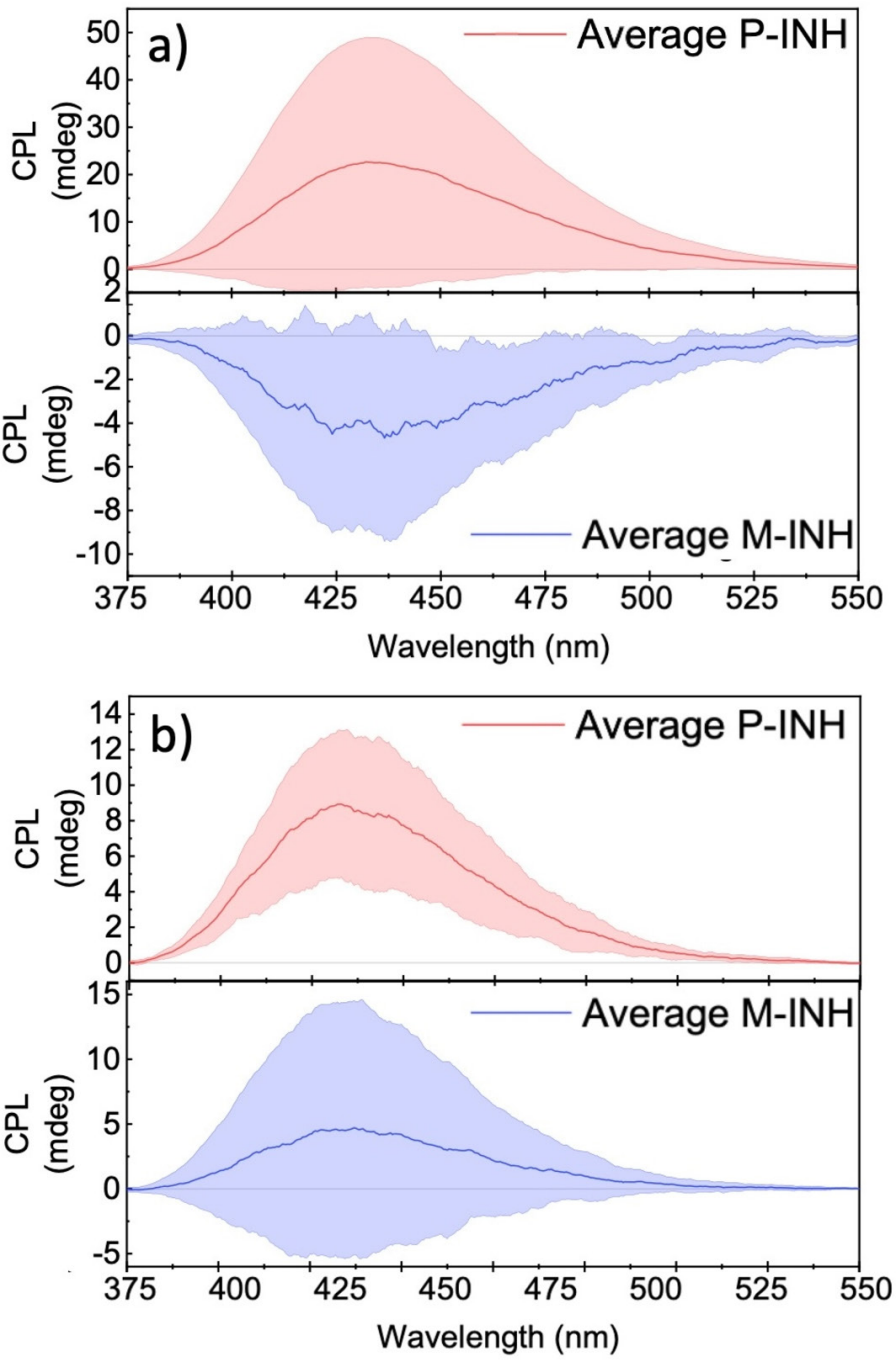
CPL and fluorescence of (a) slow‐dried (4°C) and (b) fast‐dried (20°C) TPE on *M*‐ and *P*‐SNH films. Shaded areas indicate spread of signals used in determining the average signal (colored solid line).

The CPL spectra (excitation at 333 nm) for slow‐dried (4°C) films mirrored the ECD handedness: *P*‐SNH induced positive CPL, and *M*‐SNH induced negative CPL (Figure 3a). In contrast, fast drying (20°C) yielded highly variable CPL intensities and occasional sign inversions, particularly for *M*‐SNH, indicating weaker correlation between CPL sign and SNH handedness (Figure 3b). Dissymmetry factors *g*_lum_ reached −9 × 10^−3^ for *M*‐SNH and 1.7 × 10^−2^ for *P*‐SNH—slightly higher than *g*_abs_ values and consistent with values reported by Hu et al. (1.3 × 10^−2^) [12]. Control experiments confirmed that CPL signals originated indeed from emission as rotating the sample (±90°) or flipping the substrates did not alter the intensity or sign.

**FIGURE 4:**
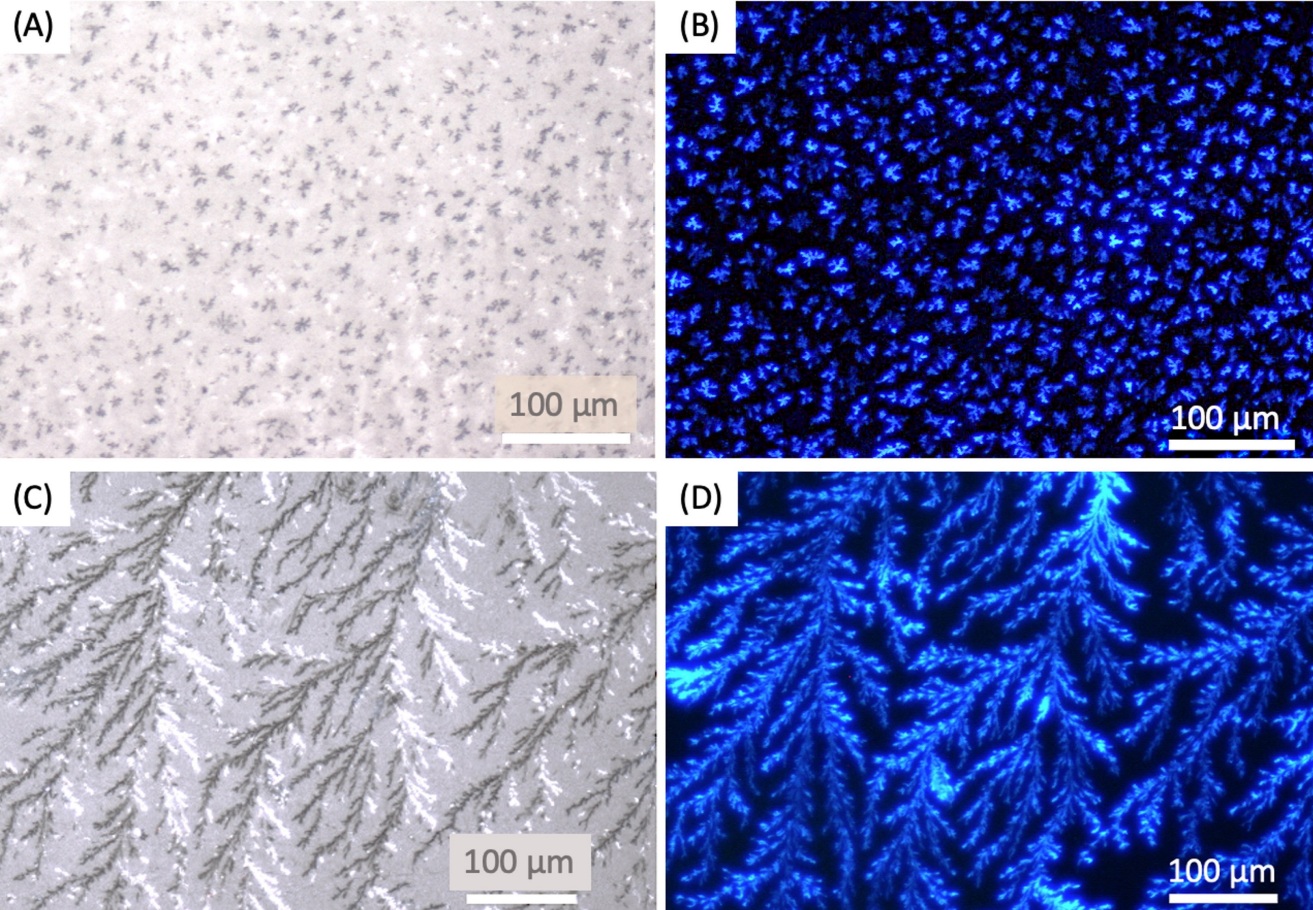
Differential interference contrast images (A, C) and epifluorescence images (B, D) of slow‐dried (A, B) and fast‐dried (C, D) TPE drop‐cast on *P*‐SNH films.

**FIGURE 5:**
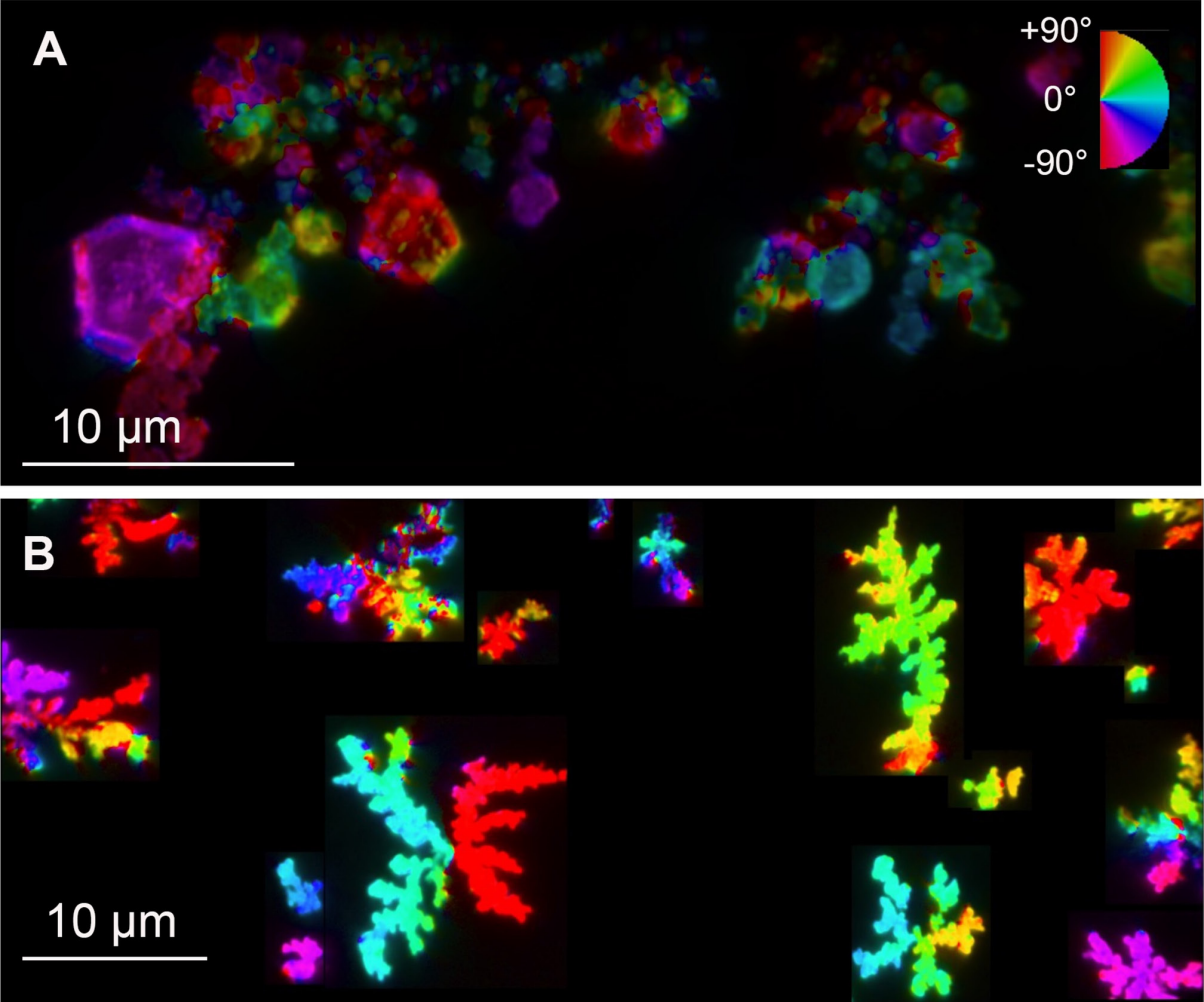
Hyperpolarimetric images of slow‐dried (A) and fast‐dried (B) TPE *P*‐SNH substrates. Colors indicate emission polarization vector azimuth angles.

Nomarski (DIC) and epifluorescence microscopy revealed distinct drying rate–dependent morphologies (Figure 4). Fast drying consistently yielded highly branched dendritic crystals, whereas slow drying produced more compact crystalline domains. For both fast‐ and slow‐dried samples, it was observed that the fluorescence emission principally originated from the crystalline domains. The orientation of the chromophores within these domains was probed using hyperpolarimetric microscopy in which the emission intensity and polarization are mapped simultaneously (Figure 5). This confirmed that the emission originates almost exclusively from crystalline domains and further evidenced that they exhibit strong linear polarization.

In the case of the slow‐dried samples (Figure 5A), we observe the formation of small (1–5 μm) crystals with regular facets and uniform linear polarization through each crystal. Their shape and angular facet orientations of ~90° and 110° are consistent with the geometry expected for orthorhombic crystals resulting from the P2_1_ space group of TPE [12, 13]. In contrast, fast‐drying samples (Figure 5B) formed dendritic aggregates of sub‐200‐nm crystallites. Interestingly, the latter also exhibited strong linear polarization across extended regions. While this is typically observed for single crystals in which all of the emitting chromophores are well oriented, it is more surprising for dendritic crystal growth where small crystalline domains may grow along different directions.

**FIGURE 6:**
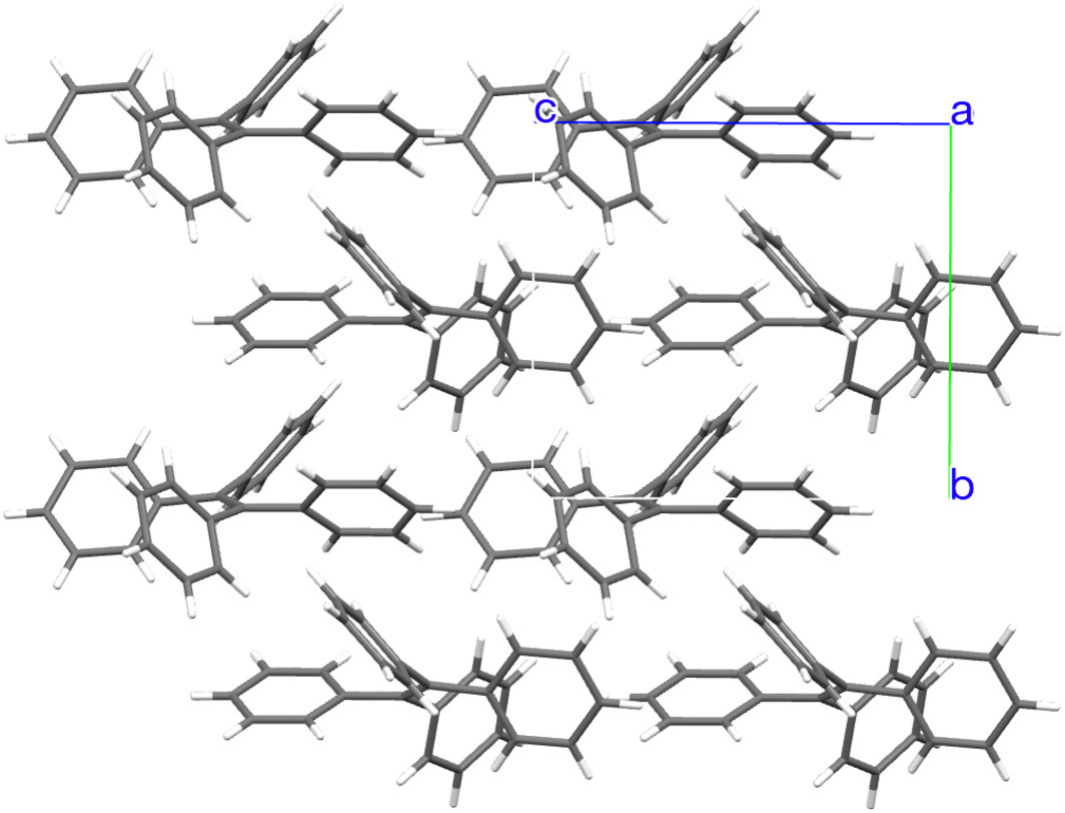
*P*2_1_ crystal lattice of TPE viewed along the *o*–*a* axis, showing collinear orientation of TPE chromophore (from Ref [41]).

In TPE crystals, the exocyclic C=C bonds are arranged collinearly, perpendicular to the (100) face (Figure 6) [41]. This induces alignment of the electronic transition dipole moments, governing light absorption and emission with respect to the crystallographic axes. Thus, the uniform polarization across both compact and dendritic crystals reflects a specific chromophore alignment that is fixed at nucleation and then preserved during crystal growth.

From the microscopy studies, we infer that higher drying temperatures result in faster solvent evaporation and accelerated crystal growth, thereby reducing the correlation between nascent crystallites and the underlying helical silica scaffold. This decoupling likely reduces chiral discrimination at the interface. In contrast, slower solvent evaporation at lower temperatures favors the preferential nucleation of crystallites at sites stabilized by interactions with the SNHs. These then induce the selective formation of crystallites with consistent chiroptical signals. For this reason, the CPL signals correlate more strongly with SNH handedness for slow‐dried samples and not with the dendritic structures obtained upon fast drying.

The ECD and CPL signs correlate consistently with SNH handedness: *P*‐SNHs induced positive ECD and CPL signals under slow‐drying conditions, whereas *M*‐SNHs induced negative signals. Direct assignment of the crystal enantiomer by single‐crystal diffraction was precluded by the small crystal size and their confinement to the SNH film surface. However, the assignment can be made by analogy with the literature. Li et al. [42] and Hu et al. [12] have assigned a positive ECD signal in the 270–350‐nm region to *M*‐helical TPE crystals. Since *P*‐SNHs consistently induce positive ECD, we conclude that *P*‐SNHs preferentially nucleate the *M*‐enantiomorph of TPE, while *M*‐SNHs preferentially nucleate the *P*‐enantiomorph.

The CPL signals observed for slow‐dried samples indicate that SNH handedness biases the sign of the chiroptical response and, by extension, the predominant helical conformation adopted by TPE chromophores within the emissive crystalline domains, although the substantial sample‐to‐sample dispersion of CPL intensities suggests that this bias is partial rather than absolute. In contrast, fast‐dried dendritic assemblies exhibit strong ECD but weak and poorly correlated CPL, consistent with rapid nucleation of small crystallites whose growth is not sufficiently influenced by the SNH template to enforce enantioselective packing.

It is important to underline the complementary nature of these two chiroptical observables. ECD probes the absorption of all TPE molecules in contact with the SNH surface, adsorbed, amorphous, and crystalline alike, and therefore reports on the supramolecular organization at the interface as a whole. CPL, in contrast, can arise only from emissive species, which in this system are in majority the crystalline domains, since molecularly dispersed and amorphous TPE remain largely non‐emissive. This emission‐based selectivity is mirrored in the optical microscopy observations: Under slow drying, the well‐faceted orthorhombic crystals that grow in close registry with the SNH film (Figures 4A,B and 5A) yield strong CPL whose sign tracks the SNH handedness, whereas the dendritic crystallites formed upon fast drying (Figures 4C,D and 5B) give weak and poorly correlated CPL despite producing comparable ECD intensities. The observation that CPL is nevertheless present and sign‐correlated with SNH handedness in slow‐dried samples therefore supports the view that the chiral silica surface biases the nucleation step itself, rather than merely organizing a chirally arranged molecular adlayer. In other words, the SNHs act as templates that enantioselectively bias crystal growth, and the agreement between ECD and CPL signs in the slow‐drying regime reflects strongly a surface‐induced enantiomeric enrichment of the crystallites in spite of the dispersion of CPL intensities even under slow drying.

## 4 Conclusion

We have demonstrated that, when drop‐cast onto substrates functionalized with an amorphous film of chiral SNHs, TPE exhibits strong induced ECD and CPL signals whose signs are highly biased by the handedness of the underlying SNH template. ECD, which reflects supramolecular organization at the SNH interface, is highly reproducible and correlates directly with SNH handedness, positive for *P*‐SNH and negative for *M*‐SNH. CPL, which reports specifically on the helical conformation of TPE within crystalline domains, while showing greater spatial variability, correlates with SNH handedness under slow‐drying conditions but becomes less correlated when rapid evaporation kinetics outpace template‐directed nucleation.

ECD and CPL thus act as complementary, nonredundant probes: ECD reports on the full population of TPE molecules adsorbed at the SNH interface, whereas CPL is emitted mainly by the crystalline domains. Their concordant sign with SNH handedness in the slow‐drying regime indicates that the chiral silica surface biases nucleation itself, supporting a surface‐induced enantioselective bias of crystallization. These results indicate that mesoscopic morphological chirality can bias molecular‐level enantiomer selection during crystallization, transmitting structural information across three orders of magnitude in length scale without covalent chiral auxiliaries. This non‐covalent, morphology‐driven chirality transfer offers a scalable and modular strategy for the enantioselective growth of luminescent chiral crystals and the bottom‐up fabrication of CPL‐active photonic materials.

## Funding

Financial support from the ANR (project no. ANR‐21‐CE09‐0012‐02, no. ANR‐23‐EXLU‐0004), Bordeaux Univ., and the CNRS is gratefully acknowledged.

## Data Availability

The data that support the findings of this study are available from the corresponding author upon reasonable request.
